# Supplementary material for: Leaving No Women Behind: Evaluating the Impact of the COVID-19 Pandemic on Livelihood Outcomes in Kenya and Ethiopia
Source: Int J Environ Res Public Health. 2023 Mar 13;20(6):5048. doi: 10.3390/ijerph20065048 (PMC10049247; doi:10.3390/ijerph20065048)
Supplement: Supplementary file 1 [file ijerph-20-05048-s001.zip › ijerph-2257849-supplementary.pdf]

Supplementary materials

Leaving no women behind: Evaluating the impact of the COVID-19 pandemic on livelihood outcomes in Kenya and Ethiopia

Marshall Makate [1,*] and Clifton Makate [2]

1 Curtin School of Population Health, Curtin University, Kent Street, Perth 6102, Australia
2 School of Economics and Business, Norwegian University of Life Sciences, P.O. Box 5003, 1432 Ås, Norway
* Correspondence: marshall.makate@curtin.edu.au; Tel.: +61-89-2266-4450

Characteristics of female-headed families in Kenya and Ethiopia

The primary findings of our paper shows that individuals living in female-headed families were disproportionately impacted by the pandemic in both Kenya and Ethiopia. To better understand these findings, Tables S1 and S2 provides a summary of the relationship between being a female-headed family and selected socioeconomic characteristics of these households before the pandemic. These results are generated using the regression model as described in the main text (Equation 1) where we estimate series of linear probability models in which each characteristic serves as the outcome variable and the indicator for female head of household is the main explanatory variable. We also include controls for age fixed effects, rural residence, and region fixed effects.

The results for both countries give a possible explanation as to why female-headed families in both countries appeared to have been impacted the most by the COVID-19 pandemic. We show that female-headed families in both countries are more likely to be classified as coming from a low socioeconomic status position compared to their male-headed counterparts.

Table S1: Relationship between being a female-headed family and the probability of having selected characteristics before the COVID-19 pandemic in Kenya.

	**No assets**		**Radio**		**Mattress**		**Charcoal Jiko**		**Refrigerator**	
	β	SE	β	SE	β	SE	β	SE	β	SE
Female-headed family	0.007	0.006	-0.066***	0.011	-0.008	0.007	-0.0002	0.011	-0.004	0.006
Observations	72684		72684		72684		72684		72684	

	Television		Landline telephone		Computer /laptop/tablet		Internet access at home		Secondary education	
	β	SE	β	SE	β	SE	β	SE	β	SE
Female-headed family	-0.128***	0.031	0.011	0.008	-0.021*	0.010	0.039***	0.006	-0.033***	0.003
Rural residence indicator	Yes		Yes		Yes		Yes		Yes	
Age fixed effects	Yes		Yes		Yes		Yes		Yes	
Region fixed	Yes		Yes		Yes		Yes		Yes	

effects					
Observations	2366	2366	2366	72684	72684

Notes: ***Significant at 1% level; *significant at 10% level. β = marginal effect. SE = standard error.

Table S2: Relationship between being a female-headed family and the probability of having selected characteristics before the COVID-19 pandemic in Ethiopia.

	**Radio**		**Television**		**Gas stove**		**Electric stove**		**Refrigerator**	
	β	SE	β	SE	β	SE	β	SE	β	SE
Female-headed family	-0.080***	0.009	-0.080***	0.009	-0.010***	0.003	-0.036***	0.008	-0.075***	0.008
Observations	17563		17563		17563		17563		17563	

	Married		Divorced/ separated/widowed		Literate		Ever attended school		Health Insurance coverage	
	β	SE	β	SE	β	SE	β	SE	β	SE
Female-headed family	-0.413***	0.011	0.489***	0.011	-0.142***	0.010	-0.123***	0.010	-0.017*	0.008
Rural residence indicator	Yes		Yes		Yes		Yes		Yes	
Age fixed effects	Yes		Yes		Yes		Yes		Yes	
Region fixed effects	Yes		Yes		Yes		Yes		Yes	
Observations	16969		16969		16964		16964		16965	

Notes: ***Significant at 1% level; *significant at 10% level. β = marginal effect. SE = standard error.

Appendix of survey questions used to create main outcome variables.

Kenya

Please note that all these questions were extracted from the original surveys for Kenya [1].

Question I

"Compared to before March 2020, before the lockdown / pandemic, are you more / less/ or equally worried about your household not having enough food?"

i. More worried
ii. Equally worried
iii. Less worried

We coded responses in which individuals had indicated to have been "*more worried*" as one and zero otherwise (i.e., equally worried + less worried).

Question II

	In the past 7 DAYS, how many days have ADULTS in your household….	In the past 7 DAYS, how many days have CHILDREN (<18) in your household….
i. Gone to bed hungry?	[-------]	[-------]
ii. Skipped meals or cut the amount of meals?	[-------]	[-------]
iii. Gone entire days without food?	[-------]	[-------]

We created binary variables reflecting "adults skipping meals", "children skipping meals" and "adults going hungry" if there was indication that anyone from the household had gone to bed without eating for at least a day or more.

Ethiopia

During the last 30 days, was there a time when You or any other person in your household were worried about not having enough food to eat because of lack of money or other resources?

During the last 30 days, was there a time when You, or any other person in your household, were unable to eat healthy and nutritious/preferred foods because of a lack of money or other resources?

During the last 30 days, was there a time when you, or any other person in your household, ate only a few kinds of foods because of a lack of money or other resources?

During the last 30 days, was there a time when you or any other person in your household, had to skip a meal because there was not enough money or other resources to get food?

During the last 30 days, was there a time when You, or any other person in your household, ate less than you thought you should because of a lack of money or other resources?

During the last 30 days, was there a time when You, or any other person in your household, went without eating for a whole day because of a lack of money or other resources?

During the last 30 days, was there a time when Your household ran out of food because of a lack of money or other resources?

During the last 30 days, was there a time when You, or any other person in your household, were hungry but did not eat because there was not enough money or other resources for food?

References

1. World Bank, U.o.C.B., Kenya National Bureau of Statistics, . *Kenya - COVID-19 Rapid Response Phone Survey with Households 2020-2022, Panel, Waves 1-8 (COVIDRS). Ref: KEN_2020_COVIDRS_v07_M.* 2021 [cited 2021 30/08]; Available from: https://microdata.worldbank.org/index.php/catalog/3774.
